# Vertical Displacement Index and Early Treatment-Related Physiological Improvement in Acute Respiratory Failure: An Exploratory Ultrasound-Based Study

**DOI:** 10.3390/arm94030040

**Published:** 2026-06-22

**Authors:** Bedriye Müge Sönmez, İlker Şirin, Gülşen Akçay, Murat Özdemir, Necip Gökhan Güner

**Affiliations:** 1 Department of Emergency Medicine, University of Health Sciences Dışkapı Yıldırım Beyazıt Training and Research Hospital, 06030 Ankara, Turkey; sirinilkerr@gmail.com (İ.Ş.); gulakcay@yahoo.com.tr (G.A.); 2 Air Ambulance Unit, Ministry of Health, 06800 Ankara, Turkey; doktoraiki@gmail.com; 3 Department of Emergency Medicine, University of Sakarya, 54187 Sakarya, Turkey; gunernecipgokhan@gmail.com

**Keywords:** respiratory failure, emergency department, lung ultrasound, vertical displacement index, early treatment-related physiological improvement

## Abstract

**Highlights:**

**What are the main findings?**

- VDI decreased significantly after initial treatment across oxygen saturation groups, while baseline VDI was higher in patients with more severe hypoxemia.
- ΔVDI was weakly associated with changes in oxygen saturation and pH; VDI decreased in pulmonary edema, COPD/asthma, and pneumonia, but not in pulmonary embolism.

**What are the implications of the findings?**

- VDI may be explored as a non-invasive bedside parameter for the early reassessment of physiological improvement in acute respiratory failure.
- VDI should be interpreted as a complementary, hypothesis-generating parameter alongside clinical and laboratory findings, pending validation in larger standardized studies.

**Abstract:**

**Objective**: Rapid assessment of early treatment-related physiological improvement in emergency department (ED) patients with respiratory failure (RF) remains challenging. Blood gas analysis is informative but invasive and not ideal for repeated use. The vertical displacement index (VDI), an ultrasound-derived parameter based on pleural motion, may provide dynamic bedside information on early physiological change. This study evaluated whether changes in VDI are associated with early physiological improvement in ED patients with RF. **Methods**: This prospective observational study was conducted in the EDs of two tertiary care hospitals. Adult patients presenting with dyspnea and clinical evidence of RF were included. VDI was measured by lung ultrasound at baseline and 30 min after initial treatment. The primary endpoint was the change in VDI 30 min after the initial treatment, calculated as the difference between pre-treatment and post-treatment VDI. The expected direction was a post-treatment decrease in VDI, with greater VDI reduction expected to be associated with greater early physiological improvement. Secondary analyses included comparisons of VDI changes across oxygen saturation and diagnostic groups, as well as correlations between ΔVDI and physiological changes. Patients were grouped by admission oxygen saturation (<80%, 80–90%, and ≥90%). **Results**: Seventy-nine patients were included. Pre-treatment VDI differed significantly between oxygen saturation groups, with the highest values in the most hypoxemic patients (*p* = 0.028). VDI decreased significantly after treatment in all groups (*p* < 0.001 for all), with the greatest reduction in the <80% group. By diagnosis, VDI decreased significantly in pulmonary edema, COPD/asthma, and pneumonia, but not in pulmonary embolism (*p* = 0.138). VDI reduction correlated positively with improvements in oxygen saturation (r = 0.27, *p* = 0.016) and pH (r = 0.24, *p* = 0.037), but not with CO_2_. **Conclusions:** VDI may be explored as a practical ultrasound-derived bedside parameter associated with early physiological improvement in ED patients with RF.

## 1. Introduction

Respiratory failure (RF) is a common and potentially life-threatening condition frequently encountered in the emergency department (ED) and requires rapid assessment and timely management [1,2]. Early evaluation of early treatment-related physiological improvement is essential for guiding clinical decision-making and optimizing patient outcomes in this setting [3,4]. Arterial blood gas (ABG) analysis is considered the reference standard for assessing respiratory status, particularly in evaluating gas exchange and acid–base balance. However, ABG sampling is invasive, painful, and not always suitable for repeated measurements during the early phase of treatment in the ED [5,6]. For this reason, non-invasive bedside tools have emerged as essential components of monitoring in patients with RF [4,7].

Pulse oximetry provides a rapid, continuous, and non-invasive assessment of oxygenation and plays a central role in the initial evaluation and follow-up of these patients [8]. Nevertheless, oxygen saturation primarily reflects oxygenation status and does not directly provide information on respiratory mechanics or the work of breathing, both of which are important components of clinical improvement [9]. Therefore, there is a need for additional bedside tools that can provide dynamic information about respiratory function beyond oxygenation alone.

In recent years, point-of-care ultrasound (POCUS), particularly lung ultrasound (LUS), has emerged as a valuable bedside tool for assessing patients with respiratory conditions. LUS enables real-time, dynamic evaluation of pulmonary pathology and has been increasingly incorporated into emergency medicine practice. However, most ultrasound-based approaches rely on qualitative findings, and quantitative parameters that reflect dynamic respiratory changes remain limited [4,10].

The vertical displacement index (VDI), adapted from the pleural vertical displacement comparison index described by Martins and Nogué, is an ultrasound-derived parameter that quantifies pleural movement during respiration and may provide insight into respiratory mechanics. From a physiological perspective, VDI is based on the cranio-caudal displacement of the pleural line during the respiratory cycle [11]. In acute RF, increased respiratory effort, altered thoracic mechanics, reduced lung aeration, bronchoconstriction, pulmonary congestion, or impaired compliance may modify pleural motion observed on M-mode ultrasound [12]. Early treatment may reduce respiratory effort, improve ventilation and lung aeration, and decrease excessive pleural displacement [13]. Therefore, a reduction in VDI after treatment may be associated with short-term improvement in respiratory mechanics. In parallel, improvements in respiratory mechanics may accompany improvements in oxygenation, although VDI should not be interpreted as a direct measure of gas exchange. However, the previous literature on VDI remains limited; it has primarily been described as a potential ultrasonographic sign associated with bronchospasm and accessory muscle recruitment, and it has not been sufficiently evaluated as a dynamic monitoring parameter in heterogeneous ED patients with acute RF [11,14]. In contrast, most established lung ultrasound applications in acute RF focus on diagnostic pattern recognition, such as B-lines, consolidation, pneumothorax, pulmonary edema, or pleural sliding [15,16,17], rather than quantifying short-term treatment-related pleural motion [12,18]. Thus, whether ΔVDI changes after initial ED treatment and whether these changes are associated with early physiological improvement remain unclear. The present study, therefore, aimed to evaluate whether treatment-related VDI change (ΔVDI) is associated with short-term physiological improvement in ED patients with acute RF.

## 2. Materials and Methods

### 2.1. Study Design and Setting

This prospective observational study was conducted in the emergency departments of two tertiary care hospitals between 1 April 2022, and 1 April 2023. Due to the renovation of the first hospital, patient recruitment continued in the second center after 1 October 2022. Ethical approval was obtained from the institutional review boards of both hospitals (Decision numbers: 118/90 and 223-336). Written informed consent was obtained from all participants prior to enrollment.

### 2.2. Study Hypothesis and Methodological Rationale

The methodological approach was structured around the central hypothesis that VDI would decrease after initial treatment and that a greater reduction in VDI would be associated with early physiological improvement. Therefore, paired baseline and 30 min post-treatment VDI measurements were used to quantify short-term within-patient change. ΔVDI, calculated as pre-treatment VDI minus post-treatment VDI, was defined as the primary measure of VDI reduction. VDI changes were compared across oxygen saturation and diagnostic subgroups to examine whether the observed treatment-related VDI reduction showed a consistent pattern across different levels of baseline hypoxemia and underlying etiologies. Correlations between ΔVDI and changes in oxygen saturation, RR, pH, and CO_2_ were then assessed, as these variables were selected as clinically relevant bedside and laboratory indicators of early respiratory and acid–base change. The study was designed to evaluate VDI as an exploratory bedside reassessment parameter, rather than as a prognostic marker for outcomes such as intubation, intensive care unit (ICU) admission, or mortality.

### 2.3. Study Population

**Figure 1:**
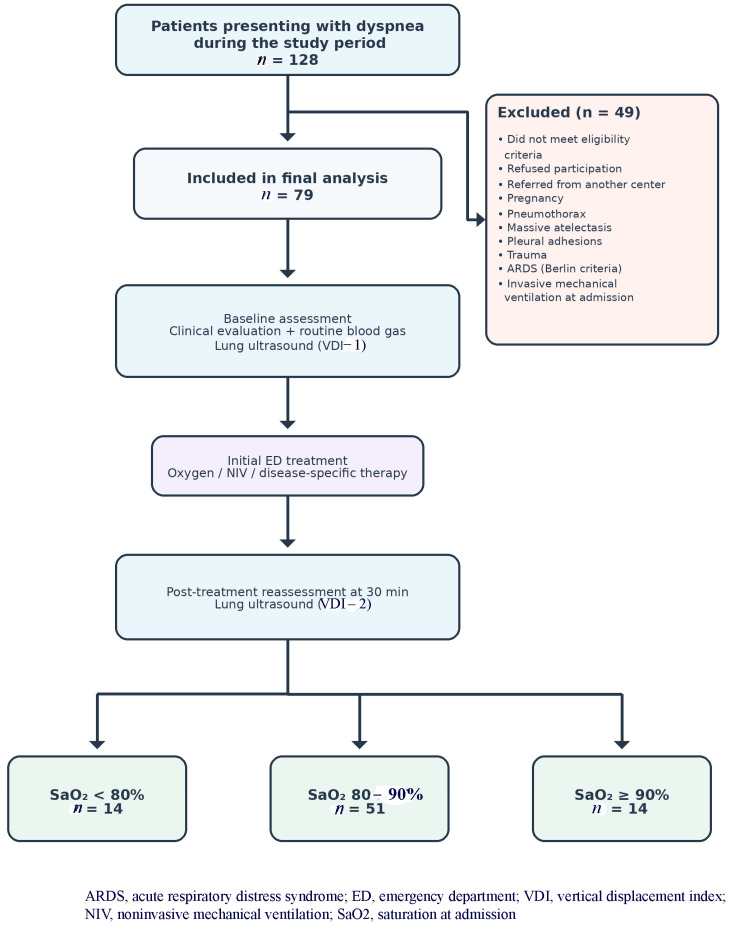
Flow chart of the study.

A priori sample size calculation using G*Power (v3.1.9) indicated that a minimum of 54 patients would be required to achieve 95% power at α = 0.05, assuming a 5% change in vertical pleural displacement. Adult patients (≥18 years) presenting to the ED with dyspnea and clinical evidence of RF were considered eligible for inclusion. Patients were excluded if they were pregnant, refused participation, were referred from another center, had pneumothorax, massive atelectasis, pleural adhesions, trauma, or were diagnosed with acute respiratory distress syndrome according to the Berlin criteria [19]. Patients requiring invasive mechanical ventilation (MV) at admission were also excluded. During the study period, 128 patients presented with dyspnea. Of these, 49 were excluded based on the predefined criteria, and 79 patients were included in the final analysis. A flow diagram summarizing patient inclusion and exclusion is presented in Figure 1.

### 2.4. Data Collection

Demographic data (age, sex, comorbidities) and clinical variables were recorded at admission, including vital signs [blood pressure (BP), heart rate (HR), respiratory rate (RR), oxygen saturation, and body temperature], physical examination findings, and laboratory results, including blood gas parameters. Blood gas parameters, including oxygen saturation, pH, and CO_2_ levels, were recorded as part of routine clinical care. Due to the real-world nature of the ED setting and extraction of routine laboratory data, the sampling type could not be reliably classified as arterial or venous for all patients. The underlying causes of dyspnea were classified into major diagnostic categories representing common etiologies of acute RF in the ED, including pulmonary edema, chronic obstructive pulmonary disease (COPD), asthma, and pulmonary embolism (PE).

### 2.5. Clinical Management and Operational Definition of Early Treatment-Related Physiological Improvement

All patients received standard ED management according to their clinical condition. Oxygen therapy was initiated via a nasal cannula, simple face mask, reservoir mask, or Venturi mask, depending on the severity of hypoxemia. Non-invasive mechanical ventilation (NIV) was applied in appropriate cases, such as acute cardiogenic pulmonary edema and hypercapnic COPD exacerbations.

Patients were stratified into three predefined groups according to admission oxygen saturation: <80%, 80–90%, and ≥90%. This stratification was used as an exploratory framework to examine VDI behavior across different levels of baseline hypoxemia. The cut-off values were not intended to represent validated clinical thresholds or VDI-specific cut-off values. Target oxygen saturation was set at ≥94%, except for patients at risk of hypercapnic RF, for whom a target of 88–92% was used. Oxygen saturation, RR, and blood gas parameters are commonly used to assess and monitor acute RF and non-invasive respiratory support [3,20,21]. Therefore, these parameters were selected as clinically relevant indicators of early treatment-related physiological improvement. However, because no externally validated composite endpoint exists for short-term VDI-based monitoring in heterogeneous ED patients with acute RF, a pragmatic, study-specific operational definition was used. Early treatment-related physiological improvement was considered present when at least two of the following directionally favorable changes were observed after initial therapy: an increase in oxygen saturation, a decrease in RR, and an improvement in pH.

Treatments were categorized into major groups according to the underlying pathophysiology. Patients with obstructive airway diseases, such as COPD or asthma, received bronchodilator-based therapy, whereas those with pulmonary edema were treated with diuretics and/or vasodilators. Anticoagulation or thrombolytic therapy was administered to patients with pulmonary embolism, while supportive oxygen therapy was provided when clinically indicated.

Given the heterogeneity of underlying conditions, treatment strategies were not standardized but were applied in accordance with current ED clinical practice.

### 2.6. Ultrasound Measurements

Lung ultrasound (LUS) examinations were performed by an emergency physician with at least 5 years of ultrasonography experience. The ultrasound operator was not involved in the primary clinical management of the patients and was not informed of the admission oxygen saturation group classification at the time of VDI assessment. Oxygen saturation group classification was performed after data collection for statistical analysis; therefore, the operator was blinded to the admission oxygen saturation group at the time of VDI assessment. The operator was also blinded to blood gas results, early assessments of physiological improvement, and final clinical outcomes. However, complete blinding to visible clinical status and oxygen delivery devices was not feasible in the ED setting. All examinations were conducted without delaying clinical management. Patients were examined in the supine position. Measurements were obtained from the anterior upper lung zones (2nd intercostal space, midclavicular line). If consolidation or absence of pleural sliding was identified at the predefined measurement site during ultrasound examination, measurements were performed from the nearest adjacent area within the same anatomical region where pleural sliding could be visualized. After identification of the pleural line and confirmation of pleural sliding (“bat sign”), M-mode imaging was used to capture pleural motion. At least three respiratory cycles were recorded for each measurement. The vertical displacement of the pleura was calculated by measuring the maximum (X) and minimum (Y) distances between the skin surface and the pleural line. The vertical displacement index (VDI) was calculated using the following formula:

VDI = [(X − Y)/X] × 100

**Figure 2:**
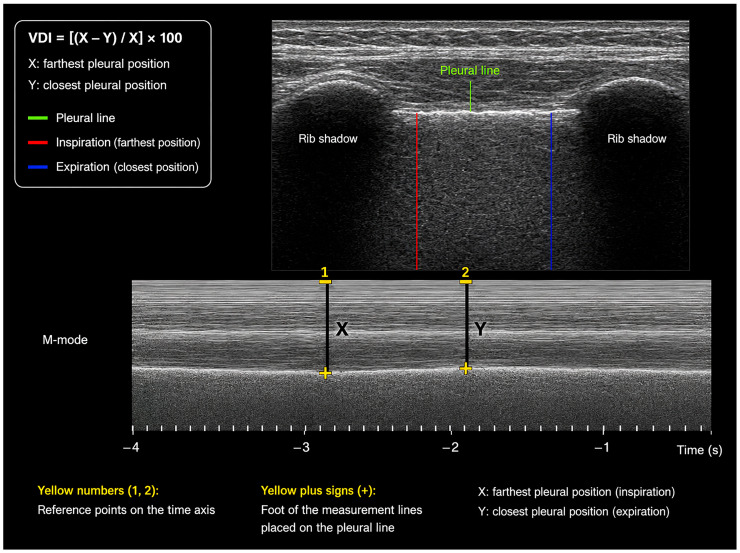
Measurement of the vertical displacement index (VDI) using M-mode lung ultrasound. VDI was calculated as the difference between pleural displacement during inspiration and expiration. The pleural line is shown with a characteristic seashore sign, and displacement was quantified between the farthest (X) and closest (Y) pleural positions over time.

Baseline (VDI-1) and post-treatment (VDI-2) measurements were obtained, with the post-treatment measurement taken 30 min after the initial treatment. A 30 min interval was selected to assess early physiological changes following initial ED management. ΔVDI was calculated as pre-treatment VDI minus post-treatment VDI; therefore, higher positive ΔVDI values indicate greater VDI reduction after treatment. An example of the measurement is shown in Figure 2.

### 2.7. Statistical Analyses

Statistical analyses were performed using SPSS software (version 21.0, IBM Corp., Armonk, NY, USA). Continuous variables were tested for normality using the Shapiro–Wilk test. As most variables were not normally distributed, data were expressed as medians and interquartile ranges (IQRs), and nonparametric tests were used. Comparisons between independent groups were performed using the Kruskal–Wallis test for continuous variables and the chi-square test for categorical variables. Within-group comparisons of pre- and post-treatment values were analyzed using the Wilcoxon signed-rank test. Post hoc pairwise comparisons were performed using the Mann–Whitney U test with Bonferroni correction (adjusted significance level *p* < 0.017). Correlations between VDI reduction and changes in physiological parameters were assessed using Spearman’s rank correlation coefficient. Bootstrap 95% confidence intervals were calculated for correlation coefficients. To assess whether oxygen saturation grouping influenced the interpretation of the findings, admission oxygen saturation was also analyzed as a continuous variable in exploratory correlation analyses with pre-treatment VDI and VDI reduction. In addition, an exploratory adjusted general linear model was performed to evaluate whether baseline hypoxemia severity and underlying diagnosis were associated with a reduction in treatment-related VDI. VDI reduction was entered as the dependent variable, baseline VDI as a covariate, and oxygen saturation group and diagnosis group as fixed factors. Partial eta-squared values were reported as effect sizes. A two-sided *p*-value < 0.05 was considered statistically significant.

## 3. Results

### 3.1. Patient Characteristics

A total of 79 patients were included in the study and categorized into three groups according to oxygen saturation at admission (<80%, 80–90%, and ≥90%). There were no statistically significant differences between the groups in age, gender, BP, laboratory parameters, or baseline clinical findings (*p* > 0.05 for all comparisons). Pre-treatment VDI values were significantly higher in patients with lower oxygen saturation levels (*p* = 0.028) (Table 1).

### 3.2. Changes in VDI According to Oxygen Saturation Groups

VDI values decreased significantly after treatment across all oxygen saturation groups, with the numerically greatest reduction observed in the most hypoxemic group (<80%). Pre-treatment VDI values differed significantly between groups, and pairwise comparisons with Bonferroni correction showed that baseline VDI was significantly higher in the <80 group than in the ≥90 group. In contrast, post-treatment VDI values and VDI reduction did not differ significantly between groups (Table 2).

To evaluate whether the oxygen saturation group findings were dependent on the predefined cut-off values, admission oxygen saturation was also analyzed as a continuous variable. Admission oxygen saturation showed a weak inverse correlation with pre-treatment VDI (rho = −0.249, 95% CI: −0.442 to −0.035, *p* = 0.027), whereas no statistically significant correlation was observed between admission oxygen saturation and VDI reduction (rho = −0.177, 95% CI: −0.374 to 0.034, *p* = 0.118).

### 3.3. VDI Changes According to Underlying Diagnosis

When analyzed by underlying diagnosis, VDI decreased significantly after treatment in patients with pulmonary edema, COPD/asthma, and pneumonia (*p* < 0.01 for all comparisons). In contrast, no significant change was observed in patients with PE (*p* = 0.138). The magnitude of VDI reduction was significantly greater in obstructive lung diseases compared to pulmonary embolism (*p* = 0.023) (Table 3).

An exploratory adjusted general linear model was performed to evaluate factors associated with treatment-related changes in VDI. Baseline VDI was included as a covariate, while baseline oxygen saturation group and diagnosis group were entered as fixed factors. In this model, baseline VDI accounted for most of the variability in VDI change after treatment, whereas the oxygen saturation group and the diagnosis group were not significantly associated with VDI change (Table 4). Treatment modality was not entered as a separate factor because it substantially overlapped with the underlying diagnostic category.

### 3.4. Changes in Clinical and Laboratory Parameters

In the overall cohort, RR and oxygen saturation improved significantly after treatment, while pH and CO_2_ also showed statistically significant but modest changes (Table 5). Because blood gas sampling could not be reliably classified as arterial or venous in all patients, findings related to pH and CO_2_ should be interpreted with caution.

### 3.5. Correlation Analysis

**Figure 3:**
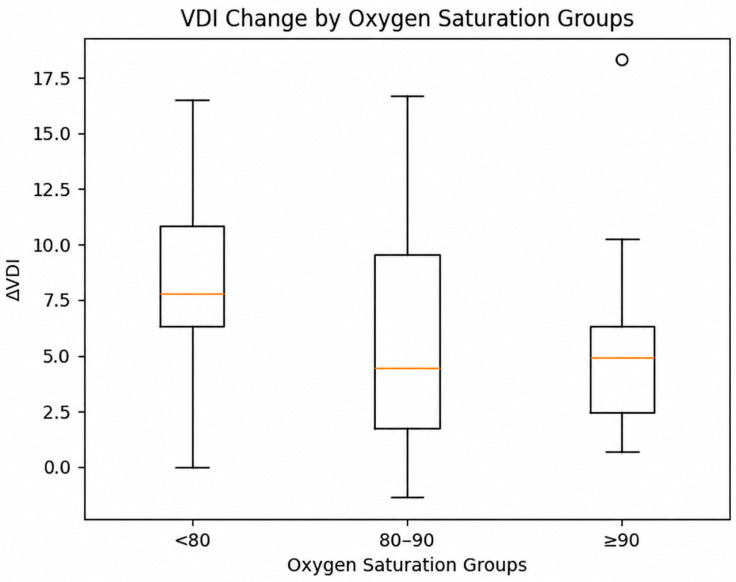
Distribution of VDI reduction across oxygen saturation groups. The greatest reduction was observed in the most hypoxemic group. VDI, vertical displacement index; ΔVDI was calculated as pre-treatment VDI minus post-treatment VDI.

**Figure 4:**
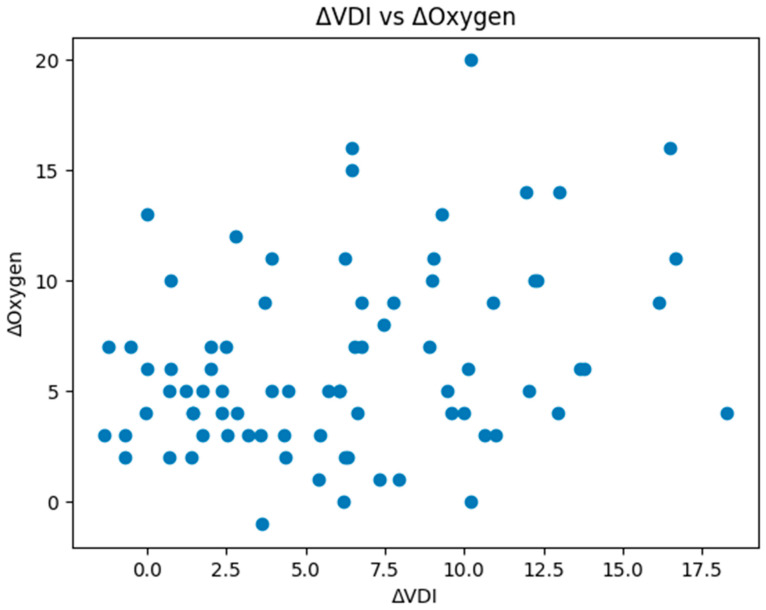
Correlation between VDI reduction and change in oxygen saturation. VDI, vertical displacement index; ΔVDI was calculated as pre-treatment VDI minus post-treatment VDI.

**Figure 5:**
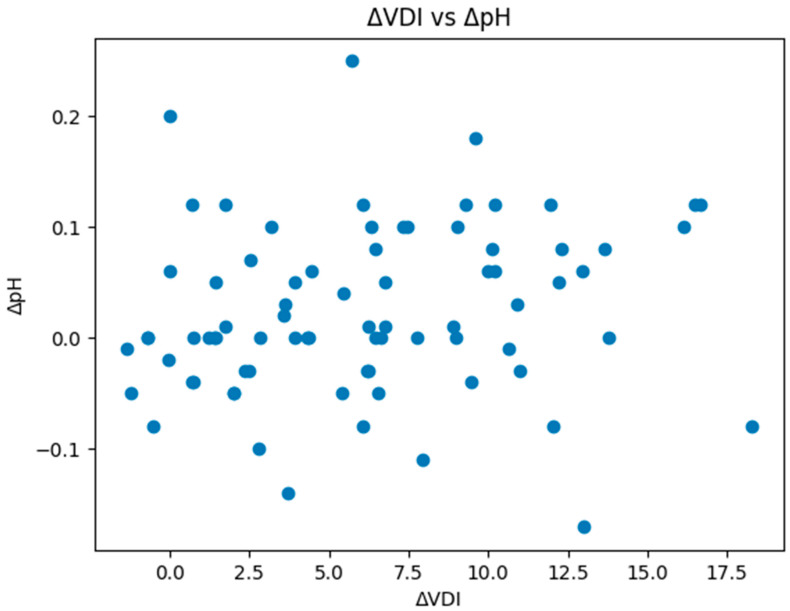
Correlation between VDI reduction and change in pH. VDI, vertical displacement index; ΔVDI was calculated as pre-treatment VDI minus post-treatment VDI.

Spearman correlation analysis is presented in Table 6. ΔVDI showed weak but statistically significant positive correlations with changes in oxygen saturation and pH, whereas its correlation with CO_2_ was not statistically significant. These findings are also illustrated in Figure 3, Figure 4 and Figure 5.

## 4. Discussion

This prospective exploratory study demonstrates three main findings. First, VDI decreased significantly following initial treatment in the overall cohort and across oxygen saturation groups. Second, ΔVDI showed weak but statistically significant associations with changes in oxygen saturation and pH. Third, a similar overall pattern was observed across different levels of initial oxygen saturation, suggesting that VDI may be associated with dynamic physiological change rather than representing a purely severity-dependent finding. Taken together, these findings support the potential exploratory role of VDI as a complementary bedside parameter of early physiological improvement. However, given the observational design, heterogeneous study population, and modest correlation magnitudes, this interpretation should remain hypothesis-generating.

Among these findings, the oxygen saturation-based analysis helps frame the clinical interpretation of VDI. The higher pre-treatment VDI observed in the most hypoxemic patients may indicate that baseline VDI is partly associated with initial hypoxemia severity. However, the absence of significant between-group differences in post-treatment VDI and ΔVDI suggests that the treatment-related reduction in VDI was not confined to any specific baseline oxygen saturation stratum. Therefore, VDI should not be interpreted as a stand-alone severity classifier. Rather, it may be considered an exploratory bedside parameter that could complement short-term reassessment when interpreted alongside clinical findings and conventional physiological measures.

This interpretation raises an important mechanistic question: which aspect of respiratory physiology might VDI be associated with? Because VDI is derived from pleural motion, it may be more closely related to changes in respiratory effort and thoracic mechanics than to gas exchange itself [22]. Pleural displacement during respiration is influenced by thoracic kinematics, including the coordinated movement of the diaphragm, chest wall, and lung. During inspiration, diaphragmatic contraction displaces the dome caudally, reduces pleural pressure, increases abdominal pressure, and contributes to outward abdominal wall motion and coupled rib cage displacement. These coordinated changes in pressure and motion provide the physiological basis for the vertical displacement of the pleural line observed on M-mode ultrasound. During respiratory distress, increased inspiratory effort and accessory muscle recruitment may further alter thoracic kinematics by increasing cranio-caudal, anteroposterior, and transverse chest wall motion, thereby augmenting ventilation [23,24]. In acute RF, pleural motion may also be influenced by the interaction between airway pressure, pleural pressure, transpulmonary pressure, and chest wall elastance. Therefore, under similar airway pressure conditions, differences in lung distension and pleural displacement may occur according to respiratory muscle load, lung recruitment–derecruitment, and chest wall mechanics. Activation of accessory, intercostal, and abdominal muscles may further modify pleural pressure and thoracoabdominal configuration, altering chest wall and abdominal displacement during inspiration. Early treatment may reduce respiratory workload, improve regional aeration or lung recruitment, decrease bronchoconstriction or congestion, and thereby modify thoracic dynamics during respiration [3,25,26,27]. Accordingly, VDI may provide complementary information on the mechanical component of early physiological improvement, while it should not be interpreted as a direct measure of oxygenation or ventilation.

While ABG analysis remains the gold standard for assessing RF in the ED [28], it is invasive and not suitable for repeated measurements or continuous monitoring. In contrast, VDI may offer a rapid, noninvasive bedside parameter that is associated with a dynamic aspect of respiratory function. In this context, our findings suggest that VDI may provide complementary, real-time information at the bedside, particularly in the early phase of treatment, and should be regarded as an adjunctive tool rather than a replacement for ABG.

How, then, should VDI be interpreted in a heterogeneous ED population? The present findings suggest that VDI should not be interpreted as a treatment- or disease-specific response parameter. In routine ED practice, patients with RF receive different therapies based on the underlying etiology, including bronchodilators, diuretics or vasodilators, anticoagulation, and supportive oxygen therapy [7]. Despite this heterogeneity, VDI showed a broadly consistent treatment-related pattern across patient groups. This observation supports the exploratory interpretation that VDI may be associated with dynamic changes in respiratory effort and early physiological improvement at the bedside, rather than representing the effect of any single intervention.

The correlation analysis provides additional support for the exploratory relevance of ΔVDI. ΔVDI showed statistically significant associations with changes in oxygen saturation and pH, suggesting that VDI reduction may vary in parallel with early physiological improvement rather than simply changing over time. However, these correlations were weak in magnitude. Therefore, ΔVDI should be interpreted as a complementary exploratory parameter rather than a stand-alone marker of early physiological improvement. This is not unexpected in a heterogeneous ED population, where RF involves multiple interacting mechanisms rather than a single physiological pathway.

The diagnosis-based analysis also provides further insight into the potential physiological meaning of VDI. Significant reductions were observed in pulmonary edema, COPD/asthma, and pneumonia, whereas no significant change was seen in PE. The absence of a significant change in patients with PE may be related to the short observation period and to the predominantly vascular nature of the disease process. Unlike conditions that more directly affect respiratory mechanics and pleural motion, PE may not produce similarly rapid treatment-related changes in pleural dynamics within the early time frame assessed in this study. Taken together, these findings suggest that VDI may be more closely associated with the mechanical aspects of respiration than with perfusion-related abnormalities.

The higher baseline VDI values and greater VDI reduction observed in obstructive airway diseases may also be considered within a broader physiological modeling framework. Systems-engineering approaches to COPD have emphasized the importance of airway resistance, lung elastance, respiratory muscle load, and integrated cardiopulmonary control in explaining disease-related respiratory behavior [29]. In this context, VDI may be viewed as a potential bedside ultrasound-derived observable that is related to the mechanical consequences of airflow limitation and altered thoracic dynamics, although this interpretation remains speculative. Future physiological or mathematical models could help clarify how changes in airway resistance, lung recruitment–derecruitment, chest wall elastance, and respiratory muscle activity influence pleural displacement and VDI dynamics. Similarly, recent machine learning approaches for differentiating causes of dyspnea in ED patients suggest that combining clinical, laboratory, and physiological variables may improve diagnostic classification [30]. VDI could potentially be evaluated as an additional dynamic ultrasound feature in such models; however, this was beyond the scope of the present study and would require larger datasets, standardized measurement protocols, and external validation.

From a practical ED perspective, these findings suggest that VDI may offer complementary real-time bedside information, particularly in settings where repeated blood gas measurements are not feasible. Its potential value may lie not in replacing existing clinical or laboratory tools, but in adding a rapid, dynamic mechanical dimension to early treatment assessment. In this sense, VDI may be particularly useful as an exploratory bedside parameter when clinicians require repeated, non-invasive reassessment of respiratory status over short time intervals.

This study has several limitations. First, treatment strategies were heterogeneous and tailored to the underlying etiology, reflecting real-world ED practice. Although this may have introduced variability in early treatment-related physiological improvement, it also enhances the external validity of the findings. In addition, variability in oxygen supplementation at the time of measurement represents a limitation, as it may have influenced oxygenation-related parameters; however, this also reflects routine clinical practice in the ED. The composite definition of early physiological improvement used in this study was not externally validated and was developed specifically as a pragmatic operational definition for the present exploratory analysis. Therefore, it should not be interpreted as a validated endpoint of treatment success. Second, blood gas measurements were obtained as part of routine clinical care, and the sampling type could not be reliably classified as arterial or venous for all patients. Because arterial and venous blood gas values are not interchangeable, particularly for CO_2_ and, to a lesser extent, pH, this may have introduced measurement heterogeneity and affected the interpretation of blood gas-related correlations. Therefore, stratified analyses according to arterial versus venous sampling could not be performed, and findings related to pH and CO_2_ should be interpreted with caution. Nevertheless, the observed weak associations may support the exploratory hypothesis that ΔVDI is related to early physiological improvement under routine ED conditions; however, this requires confirmation in studies with standardized blood gas sampling. Third, treatment protocols were not standardized, and the study did not include comparisons with established clinical severity scores, which may limit the interpretation of the clinical impact of VDI changes. The study did not evaluate associations between ΔVDI and downstream clinical outcomes such as need for intubation, ICU admission, or mortality. Therefore, the present findings should not be interpreted as evidence for the prognostic value of VDI. Future studies with larger cohorts and outcome-focused designs are needed to determine whether VDI changes have prognostic significance. Fourth, Interobserver and intra-observer reliability analyses were not performed, which represents an important limitation for an operator-dependent ultrasound-derived parameter such as VDI. Although all measurements were performed by a single experienced operator who was blinded to blood gas results and early assessments of physiological improvement, this does not replace formal reproducibility testing. Future studies should assess both interobserver and intra-observer reliability before VDI can be recommended for broader clinical use. Finally, the relatively small sample size, unequal group sizes, and exclusion of mechanically ventilated patients may limit the generalizability of the results, particularly in more critically ill populations. Despite these limitations, the present findings support the interpretation of VDI as a dynamic, clinically relevant bedside parameter that reflects early treatment-related physiological improvement in patients with respiratory failure. Accordingly, the present results should be regarded as exploratory and hypothesis-generating rather than definitive evidence for the clinical application of VDI.

## 5. Conclusions

In this exploratory study, VDI showed dynamic changes following treatment and was associated with early treatment-related physiological improvement in patients with acute RF. To our knowledge, this is one of the first prospective ED-based studies to evaluate short-term treatment-related VDI dynamics in patients with acute RF. By focusing on paired pre- and post-treatment VDI measurements, the present study adds preliminary evidence on the potential use of VDI as a dynamic bedside ultrasound parameter during the early treatment phase. However, the present results should be regarded as preliminary and hypothesis-generating rather than definitive evidence for clinical application. Further studies with larger cohorts, standardized treatment pathways, reproducibility assessments, and clearly defined physiological reference measures are needed to better clarify the potential clinical role of VDI in the early monitoring of acute RF.

## Figures and Tables

**Table 1:** Baseline characteristics and VDI values according to oxygen saturation groups.

Variable	<80 (*n* = 14)	80–90 (*n* = 51)	≥90 (*n* = 14)	*p*-Value
Age (median, IQR) Gender, *n* (%)	71.5 (62.3–86.5)	74 (64–81)	76.5 (63–84.3)	0.679
Female	6 (42.9)	31 (60.8)	8 (57.1)	0.487
Male	8 (57.1)	20 (39.2)	6 (42.9)	
SBP (median, IQR)	138.5 (82.5–156.3)	135 (110–160)	121.5 (117.5–132.5)	0.362
DBP (median, IQR)	80 (63.8–92)	80 (70–90)	70 (60–80)	0.077
Rales, *n* (%)	12 (85.7)	31 (60.8)	7 (50)	0.121
Rhonchi, *n* (%)	2 (14.3)	19 (37.3)	3 (21.4)	0.184
Creatinine (median, IQR)	1.86 (1.15–3.11)	1.16 (0.89–1.6)	1.07 (0.76–1.82)	0.280
pH (median, IQR)	7.33 (7.23–7.39)	7.35 (7.30–7.39)	7.38 (7.32–7.41)	0.176
CO_2_ (median, IQR)	39 (35.5–58.8)	41 (32.8–51)	40 (33.8–45.5)	0.813
VDI pre-treatment	13.2 (12.2–14.5)	9.1 (7–13.9)	9.3 (6.1–12.4)	0.028
VDI post-treatment	5.3 (3.1–6.9)	4.5 (3.1–6.4)	3.7 (2.8–5.9)	0.172
VDI change	7.8 (6.3–10.5)	4.3 (1.6–9.2)	4.9 (2.1–6.4)	0.124

Data are presented as median (interquartile range [IQR]) or number (%), as appropriate. Kruskal–Wallis test was used for continuous variables, and chi-square test was used for categorical variables. SBP, systolic blood pressure; DBP, diastolic blood pressure; VDI, vertical displacement index; CO_2_, carbon dioxide. ΔVDI was calculated as pre-treatment VDI minus post-treatment VDI.

**Table 2:** Within-group and between-group comparison of VDI values according to oxygen saturation groups.

Group	Pre-Treatment, Median (IQR)	Post-Treatment, Median (IQR)	Within-Group *p*-Value *	VDI Reduction, Median (IQR)
<80	13.2 (12.2–14.5)	5.3 (3.1–6.9)	0.001	7.8 (6.3–10.5)
80–90	9.1 (7.0–13.9)	4.5 (3.1–6.4)	<0.001	4.3 (1.6–9.2)
≥90	9.3 (6.1–12.4)	3.7 (2.8–5.9)	0.001	4.9 (2.1–6.4)
Between-group *p*-value †	0.027	0.173		0.124

* Within-group comparisons were performed using the Wilcoxon signed-rank test. † Between-group comparisons were performed using the Kruskal–Wallis test. Post hoc pairwise comparisons were performed using the Mann–Whitney U test with Bonferroni correction (adjusted significance level *p* < 0.017).

**Table 3:** Changes in VDI according to the underlying diagnosis.

Diagnosis	Pre-Treatment	Post-Treatment	Within-Group *p*-Value	VDI Reduction
Pulmonary edema	10.3 (7.6–13.9)	4.9 (3.4–7.2)	<0.001	6.1 (1.8–9.5)
COPD/asthma	14 (10.3–17.2)	3.8 (3.1–6.5)	0.001	9.0 (5.7–12.3)
Pneumonia	8.3 (6–13.5)	4.2 (2.7–6.4)	<0.001	5.4 (2.2–9.3)
Pulmonary embolism	8.1 (5.5–10.5)	6.7 (3.6–7.9)	0.138	0.7 (0–4.9)

Values are presented as median (interquartile range [IQR]). Pre- and post-treatment comparisons within each diagnostic group were performed using the Wilcoxon signed-rank test. Between-group comparisons were performed using the Kruskal–Wallis test. COPD, chronic obstructive pulmonary disease; VDI, vertical displacement index.

**Table 4:** An exploratory adjusted general linear model for factors associated with the change in VDI after treatment.

Source	F	*p*-Value	Partial η^2^
Baseline VDI	249.035	<0.001	0.776
Oxygen saturation group	0.232	0.793	0.006
Diagnosis group	2.103	0.107	0.081

Values were obtained from an exploratory adjusted general linear model. VDI reduction was entered as the dependent variable. Baseline VDI was included as a covariate, while baseline oxygen saturation group and diagnosis group were entered as fixed factors. Treatment modality was not included as a separate factor because it was highly collinear with diagnostic category. Partial η^2^ represents the effect size. VDI, vertical displacement index.

**Table 5:** Changes in clinical and laboratory parameters.

Parameter	Pre-Treatment	Post-Treatment	*p*-Value
Respiratory rate	24 (20–30)	18 (16–22)	<0.001
Oxygen saturation	85 (82–89)	92 (90–93.5)	<0.001
pH	7.36 (7.30–7.40)	7.40 (7.32–7.42)	0.007
CO_2_	40 (34–50.75)	38 (34–44)	0.005

Values are presented as median (interquartile range [IQR]). Comparisons between pre- and post-treatment values were performed using the Wilcoxon signed-rank test. For CO_2_, *p*-values varied across oxygen saturation groups. CO_2_, carbon dioxide.

**Table 6:** Correlation analysis between changes in VDI and physiological parameters.

Variable	Spearman’s Rho	95% CI	*p*-Value
ΔSpO_2_	0.272	0.060 to 0.463	0.016
ΔpH	0.236	0.018 to 0.459	0.037
ΔCO_2_	−0.206	−0.421 to 0.015	0.070

Values represent Spearman’s rank correlation coefficients with bootstrap 95% confidence intervals. ΔVDI was calculated as pre-treatment VDI minus post-treatment VDI; therefore, higher positive ΔVDI values indicate greater VDI reduction after treatment. CO_2_, carbon dioxide; VDI, vertical displacement index.

## Data Availability

The datasets used and/or analyzed during the present study are available from the corresponding author on reasonable request.
